# An Efficient Violet Amplified Spontaneous Emission (ASE) from a Conjugated Polymer (PFO-co-pX) in Solution

**DOI:** 10.3390/ma10030265

**Published:** 2017-03-07

**Authors:** Sara Abdulaziz Alfahd, Saradh Prasad Rajendra, Wafa Al-Mujammi, Durairaj Devaraj, Vadivel Masilamani, Mohamad Saleh AlSalhi

**Affiliations:** 1 Department of Physics, College of Science in Zulfi, Majmaah Univesrity, Az Zulfi 15981, Riyadh Province, Saudi Arabia; s.alfahd@mu.edu.sa; 2 Department of Physics and Astronomy, College of Science, P.O. Box 2455, King Saud University, Riyadh 11451, Saudi Arabia; saradprasad@gmail.com (S.P.R.); walmujammi@ksu.edu.sa (W.A.-M.); malsalhy@gmail.com (M.S.A.); 3 Research Chair on Laser Diagnosis of Cancers, Department of Physics, College of Science, P.O. Box 2455, King Saud University, Riyadh 11451, Saudi Arabia; 4 Department of Electrical and Electronics, College of Engineering, Kalasalingam University, Anad Nagar, Krishnankoil, Pin code 626190, Virudhunagar district, Tamil Nadu, India; ddevaraj.klu@gmail.com

**Keywords:** conjugated polymer PFO-co-pX, amplified spontaneous emission (ASE) spectra, single ASE peak

## Abstract

The optical of conjugated polymer poly[(9,9-dioctylfluorenyl-2,7-diyl)-co-(2,5-p-xylene)] also known as [(PFO-co-pX), ADS145UV], dissolved in a few solvents, has been measured. The absorption, emission spectra, and quantum yield have been investigated by using a spectrophotometer and spectrofluorometer, respectively. The properties of Amplified Spontaneous Emission (ASE) of conjugated PFO-co-pX polymer under different conditions such as solvent type, concentration, and pumping energy have been examined by using the tripled frequency of a Nd:YAG laser as a source of pumping. The relation between output energy and pumping energy for the samples with different concentrations in three solvents has been studied. In addition, efficiency and optical gain from the ASE were measured. Additionally, the stability of this polymer as a laser material was investigated. Among the host of conjugated polymer lasers obtained by optical pumping, this conjugated polymer has exhibited only one ASE band under a wide range of concentrations and pump power values. This is most likely due to the steric hindrance by the copolymer segment pX (2,5-p-xylene). This investigation has provided new insights into the excited state dynamics of conjugated polymer materials and has shown that this new conjugated polymer is quite efficient in the violet region.

## 1. Introduction

Laser is a very versatile instrument that has applications in every aspects of life [1,2], science [3] and technology [4]. Many materials excite laser action, like fluorescent dyes [5,6], quantum dots [7,8,9] and conjugated polymers. Conjugated polymers have been the topic of tremendous theoretical and experimental study due to its favorable uses in optical and electronic devices [10,11]. Numerous conjugated polymers have properties such as high electroluminescence efficiency, ultra-minimal operating voltage, decent mechanical elasticity, and simplicity of assembly. The photo-physical properties of conjugated polymers are yet to be fully understood. However, in recent years, conducting polymers have emerged as a smart new gain medium for lasers [12,13,14,15,16,17,18,19], solar cells [20], and optical amplifiers [21] that are tunable all through the visible spectrum.

Once the initial article of a light-emitting diode (LED) based on poly(p-phenylenevinylene) (PPV) in 1990 was published [22], countless conjugated polymers have been synthesized and reported to exhibit electroluminescence (EL) and lasers from red to blue [23]. Conjugated polymers are particularly versatile because their physical properties (color, emission efficiency) can be fine-tuned by manipulation of their chemical structures [24].

As a common simplification, a conjugated polymer is a polymer mostly with alternating single and double bonds, and if the polymer backbone does not have this alternation, it is not a conjugated polymer. There are three main families of polymers that have been studied in the context of lasers [25,26,27,28]. Among them, properties and LED applications of fluorine-based conjugated polymers and co-polymers have been highlighted in recent reviews [29,30,31].

In this paper, the spectral properties of one such conjugated polymer poly[(9,9-dioctylfluorenyl-2,7-diyl)-co-(2,5-p-xylene)] (PFO-co-pX) in THF with different concentrations are described. The results show that, under sufficient concentration and pump power, this conjugated polymer could exhibit fluorescence and laser-induced fluorescence (LIF) at 410 nm and amplified spontaneous emission (ASE) at 415 nm, with a full width half maximum (FWHM) of 4 nm with a conversion efficiency of 3.5%.

## 2. Experimental Section

**Figure 1:**
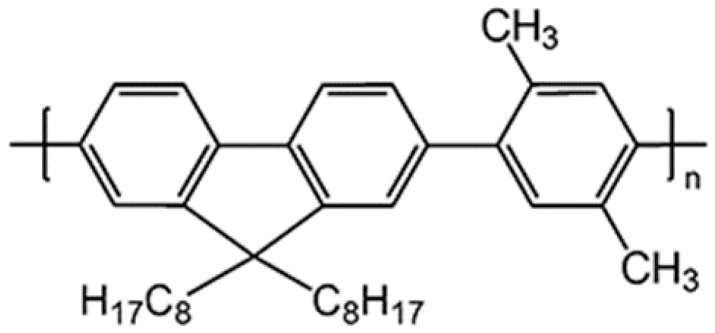
Molecular structure of conjugated polymer PFO-co-pX (ADS145UV).

A Canadian supplier, American Dye Source provided the conjugated-polymer poly[(9,9-dioctylfluorenyl-2,7-diyl)-co-(2,5-p-xylene)] (PFO-co-pX) and was used without any further purification. The macromolecule PFO-co-pX had molecular mass of 120,000 GPa (according to the data sheet). Thin layer chromatography (TLC) was done to ensure that the purity was greater than 98%. Figure 1 shows the molecular structure of the macro molecule PFO-co-pX; it was dissolved in different in spectroscopic grade solvents such as toluene, benzene, and tetrahydrofuran (THF) and also in different concentrations. 

A quartz cuvette of size 1 × 1 × 4 cm^3^, with a 1 cm path length was used to keep solutions to perform all spectral experiments. The Perkin Elmer lambda 950 spectrophotometer was used to measure absorption spectra. And a Perkin Elmer LS 55 spectrofluorometer was used to measure fluorescence spectra at room temperature; excitation wavelength was 355 nm for all solutions.

The laser used as the excitation source for all experiment was a frequency tripled (355 nm) Nd:YAG laser with a pulse width of 5 ns (Brilliant B of Quantel). The quartz cylindrical lens with a focal length of 5 cm was used to focus laser pulse. The PFO-co-pX solution kept in a four-side polished quartz cell canted (to avoid feedback) was transversely excited; for more details about the experimental setup See [32]. When the concentration of PFO-co-pX and pump energy of laser were suitably adjusted the amplified spontaneous emission (ASE) with 5 mr divergence was produced and that was collected by a fiber and fed to a spectrometer (USB4000-XR1-ES, Oceanoptics, 830 Douglas Ave, Dunedin, FL, USA). To observe the time evolution of PFO-co-pX, an ultrafast Si photodetector (UPD) (P/N:UPD-200-UP, Rise time: 120 ps, ALPHALAS GmbH, Goettingen, Germany) connected to a sampling oscilloscope (DPO4104B-L, Digital Phosphor: 1 GHz, 5 GS/s, 4 channels, Tektronix Inc., Beaverton, OR, USA), with a combined time resolution of 200 ps, was used.

## 3. Results

### 3.1. Spectral Properties

**Figure 2:**
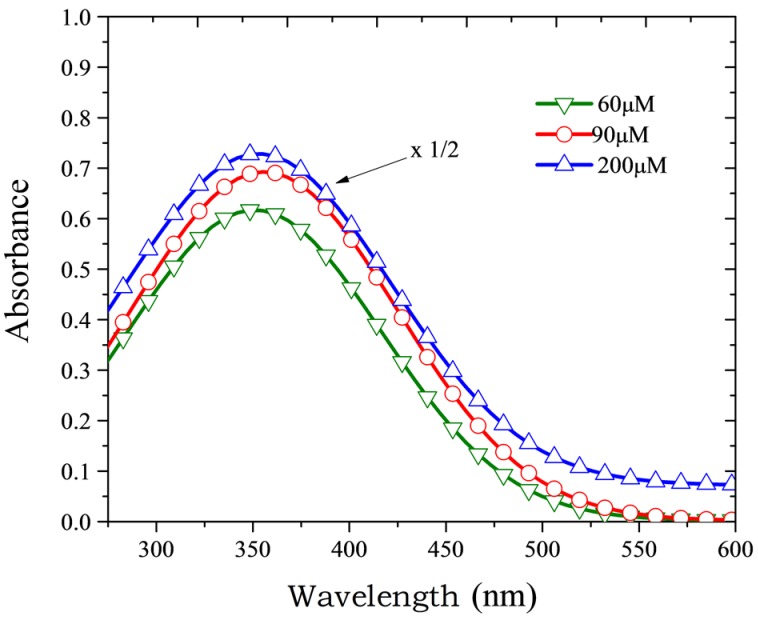
Absorption spectra of PFO-co-pX in tetrahydrofuran (THF) for different concentrations.

Figure 2 shows the absorption spectra of PFO-co-pX in THF at room temperature for different concentrations. It is clear from the figure that there is only one absorption band in the UV region around 350 nm. The shape of the absorption spectra remained the same (though the optical density increased) with increasing concentration of PFO-co-pX, indicating the absence of aggregation in the ground state [33].

**Figure 3:**
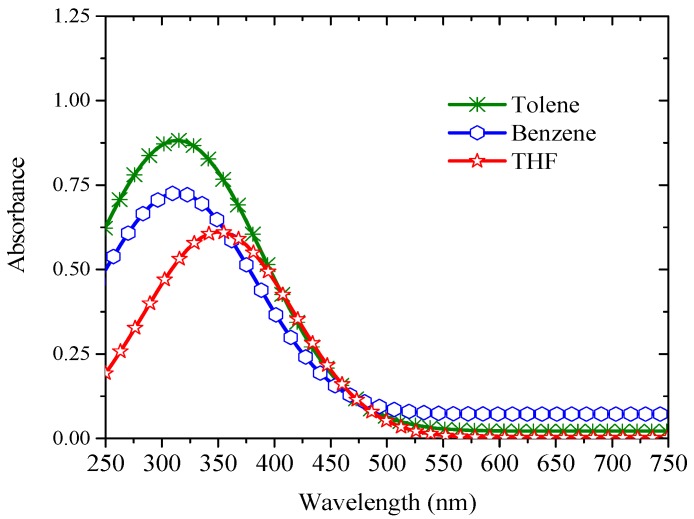
Absorption spectra of PFO-co-pX in different solvents such as toluene, THF, and benzene at a 90 μM concentration.

The absorption spectra in other solutions exhibited very similar behavior; all of them are shown in Figure 3. The shifts in the absorption peaks are due to the changes in the dielectric constant of the solvent environments.

**Figure 4:**
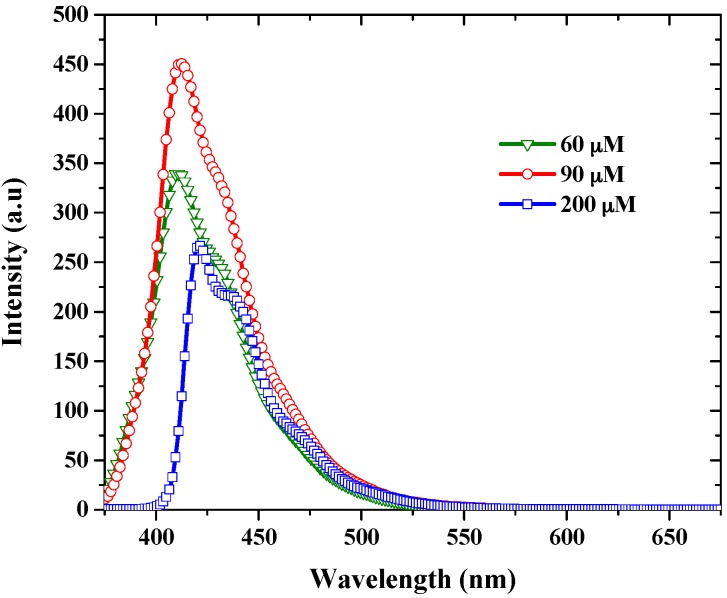
Fluorescence spectra of PFO-co-pX in THF for different concentrations.

Figure 4 illustrates the emission spectra of PFO-co-pX in THF for different concentration; again, there was only one fluorescence band around 405 nm. We had observed very small change in fluorescence spectra for PFO-co-pX in all of the above solutions, the only difference being a small shift in peak of emission wavelength. The fluorescence spectra of PFO-co-pX in high concentration solutions were slightly red shifted compared with the low concentrations in all solutions.

The Stokes’ shift (cm^−1^) of different solvent such as toluene, THF, and benzene for concentrations from 60 µM to 0.5 mM were also measured. The Stokes’ shift (cm^−1^) of the THF solution for 60 µM was 3938.3 cm^−1^, whereas it was 3571.4 cm^−1^ at 200 µM, indicating a reduction in strokes’ shift by 366 cm^−1^, due to the reabsorption process at higher concentrations. Additionally, we had observed an increase in stroke shift as the dielectric constant of the solvent environment increased.

In a series of papers reported by this group, ASE has been observed from excimeric [34], double excimeric [35], and dimeric states [36] of polymer. In contrast, this particular conjugated polymer does not form any new band in absorption or emission spectra and is distinctly different from spectral profile of many other conjugated polymers. The unchanged spectral profile of absorption and emission spectra could mean that the spectral behavior of this polymer arises from one single excited state species.

In order to investigate the temperature-dependent spectral properties, a 60 μM solution in THF was kept at various temperatures ranging from 8 °C to 50 °C, and absorption and fluorescence spectra were recorded. The results showed no significant change in absorption and fluorescence spectral profiles, though the intensities decreased for higher temperatures. Table 1 shows the quantum yield of PFO-co-pX dissolved in different solvents under the same concentration. The above set of yields were measured using Coumarin 450 in methanol as a standard [36].

### 3.2. Amplified Spontaneous Emission Properties of PFO-co-pX

Many organic laser dye like rhodamine or coumarin are dissolved in an organic solvent like ethanol and pumped with an excitation source like flash light or laser [37]. The photoactive dye absorbs photons very efficiently from an excitation source, and produce spontaneous emission. Any of the spontaneously emitted photon will induce stimulated emission and, the gain of media is so high that this produce a chain of stimulated emission and gets amplified into a super intense radiation called super radiant laser or amplified spontaneous emission (ASE). All medium that produce ASE, will produce laser, if placed in a proper cavity resonator. But not all media, which produce laser in cavity mode will produce ASE. (e.g., He-Ne laser).

**Figure 5:**
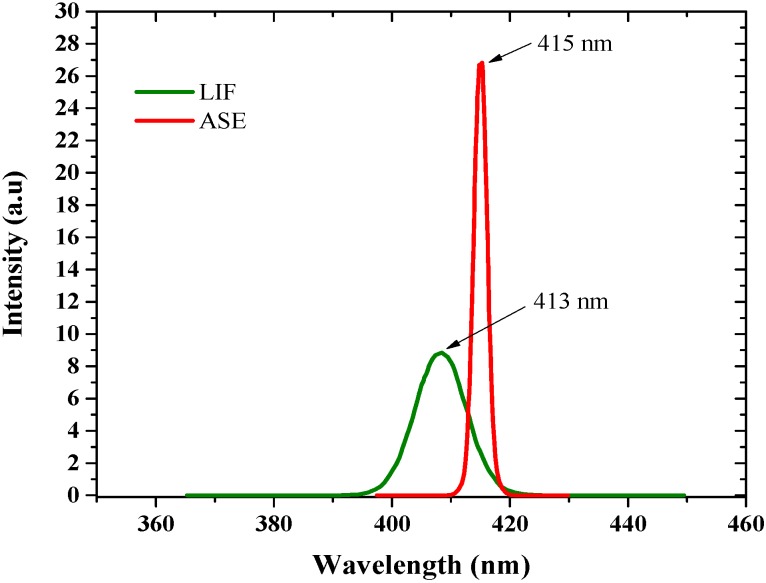
ASE Spectrum of PFO-co-pX in THF.

To investigate the laser and ASE properties of the conjugated-polymer PFO-co-pX, when excited with laser. PFO-co-pX solution of 70 μM concentration in THF was used. When a pump energy was 2 mJ, laser induced fluorescence (LIF) was produced with a peak at 413 nm, with a full width half maximum (FWHM) of 35 nm. When the pump energy was increased to 12 mJ, ASE was produced with FWHM of 6 nm with a peak at 415 nm as shown in Figure 5. 

**Figure 6:**
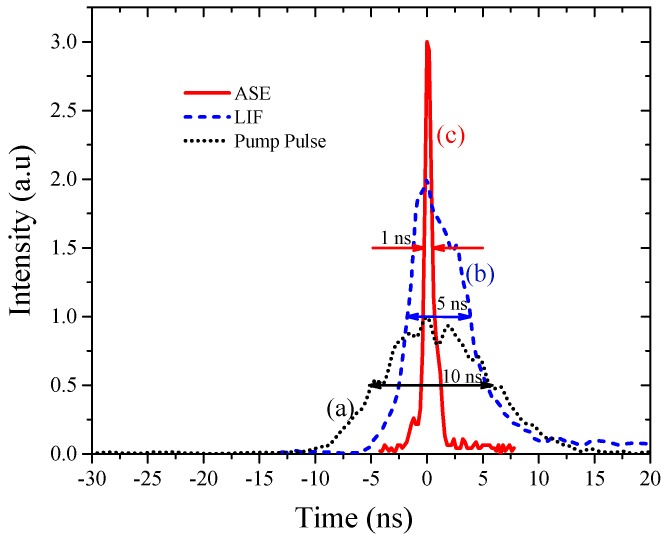
Temporal profile of ASE, LIF (PFO-co-pX), and pump pulse.

In order to monitor the temporal profile of LIF and ASE from this violet-conjugated polymer laser, the emissions were put into a UPD connected to an oscilloscope, and the trigger signal was obtained from a Q switch out of the pump laser. Figure 6a shows the temporal profile of the pump laser at 355 nm. This had the pulse width of 10 ns, with a smooth bell-shaped profile. Figure 6b shows the pulse shape of LIF, which had a pulse width of only 6 ns, with a rise time of 1 ns and a fall time of 5 ns. This was obtained for a solution with a 90 μM concentration and a pulse energy of 1 mJ.

Note that the LIF profile does not represent the true excited state life time of violet-conjugated polymer, since the sample was excited by a laser pulse of 10 ns pulse duration, and the exited state lifetime of the conjugated polymer may be less than 5 ns.

Figure 6c shows the temporal profile of the same solution, but for the pump pulse energy of 5 mJ corresponding to an irradiance of 0.66 MW/m^2^. This was well above the threshold level of ASE; when pump irradiance was high enough, an intense ASE of pulse duration 1 ns was obtained. Thus, for pump pulse energy greater than the threshold level, LIF was transformed into ASE with collimation (from 4π to 10 mr), spectral narrowing (from 30 nm to 3 nm), and temporal narrowing (from 6 ns to 0.7 ns), displaying a strong interaction between photons and inversion.

**Figure 7:**
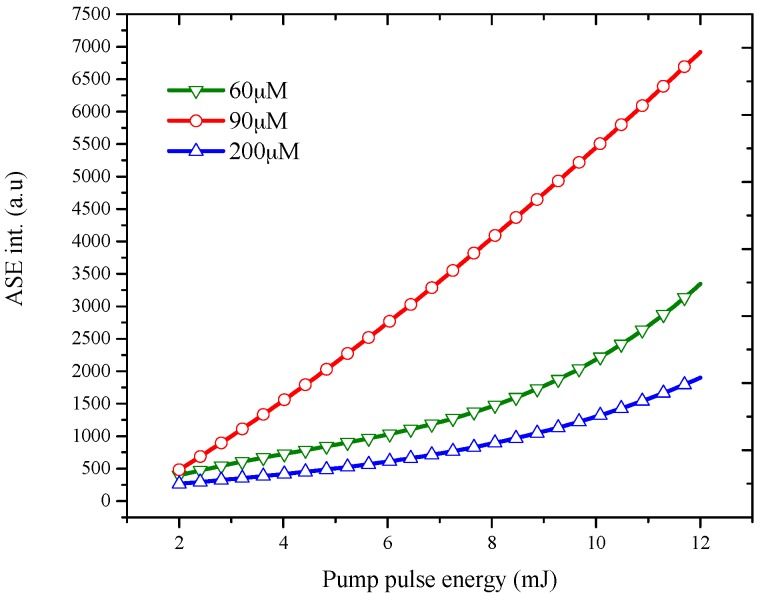
ASE intensity of PFO-co-pX dissolved in THF at different concentration for different pumping energy.

The dependence of ASE intensity on pumping energy at different concentrations were measured and are given in Figure 7. The concentrations were taken from 60 μM to 200 μM. Figure 7 shows the increase of ASE intensity of PFO-co-pX along with the increase in the pumping energy at different concentrations in THF. This is expected because high power produces high photon density and increases population inversion. The effect of concentration can be seen in Figure 7: for concentrations below 60 μM, ASE could not be observed. When the concentration was between 60 μM to 90 μM, and the ASE intensity increased as the concentration increased; this is the optimal range of concentration to produce efficient ASE. When concentration increased further, the ASE intensity started to decrease due to quenching and reabsorption losses.

**Figure 8:**
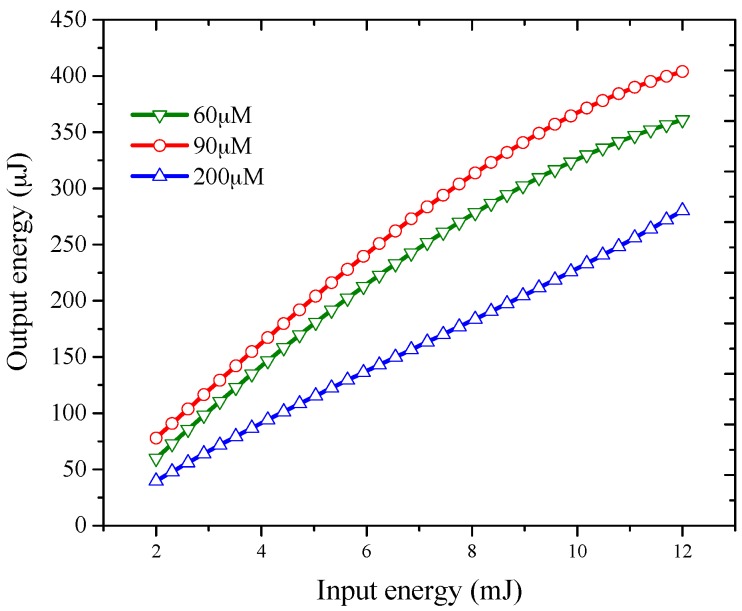
Efficiency of ASE with input energy (mJ) verses output energy (μJ).

Table 2 summarizes the maximum ASE intensity of PFO-co-pX for all solvents with a fixed concentration of 200 μM and an energy of 12 mJ. Among the solvents used, toluene exhibited an intensity that was greater than other solvents. In order to assess the efficiency of the conjugated polymer, the output of ASE was measured for a different pump power energy as shown in Figure 8. The conjugated polymer exhibited a high efficiency of 3.5% in toluene at 90 μM for a pump energy of 10 mJ.

### 3.3. The Stability of ASE Spectra

**Figure 9:**
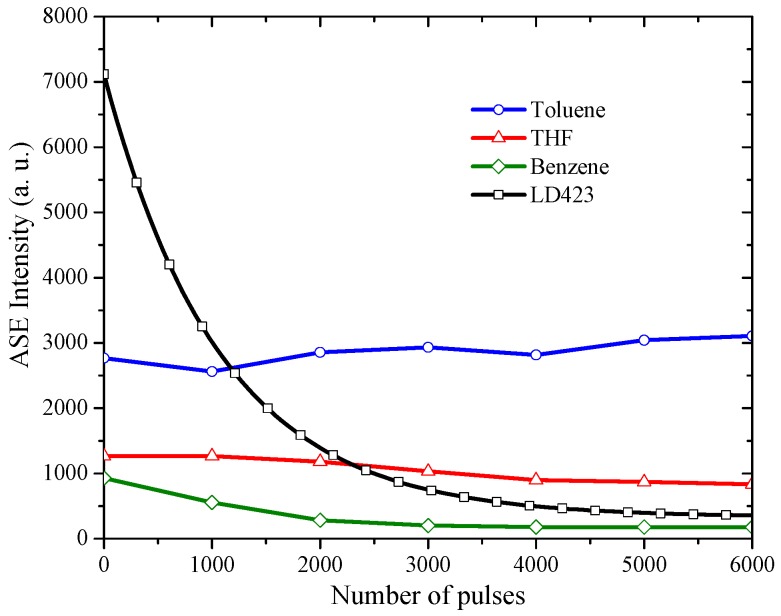
The stability of the ASE intensity versus the number of pulses for PFO-co-pX in different solvents at a concentration of 60 μM and a pump energy of 6 mJ; 0.22 M LD423 and a 6 mJ pump laser.

The stability of PFO-co-pX was studied here comparing with 1,2,3,8-tetrahydro-1,2,3,3,5-pentamethyl-7H-pyrrolo[3,2-g]quinolin-7-one (LD423). Figure 9 shows PFO-co-pX in different solvents at a concentration of 60 μM and LD423 in methanol at a concentration of 0.22 mM. The pump laser was 6 mJ. The results show that PFO-co-pX in THF and toluene were more stable than LD423 but PFO-co-pX in benzene has low stability; the solution becomes brownish after some time.

## 4. Discussion

Conjugated polymer materials are a new class of laser material that has been proven to be better than the conventional organic dye laser molecules such as coumarin, fluorescein, and rhodamines. The former ones have large stroke shifts, enabling an optically pumped laser from a high concentration in a liquid solution or solid state. Even though organic dye lasers in solid have not be shown to be as popular, conjugated polymers in a solid state are expected to become more useful over time [38].

Conjugated polymers have a strong tendency to exhibit aggregation in ground states or excited states. Recent investigations have shown ASE and lasers from excimers, double excimers, and dimeric states of some conjugated polymer materials [34,35], a characteristic hardly known in conventional laser dyes. The excitation of the molecule can lead to significant delocalization in the conjugated polymers (as evidence by large strokes’ shifts) compared to conventional dyes. This could be the main cause of the tendency towards aggregation.

In this context, the results of this report provides new insight into excited state photo-physics. PFO and PFO-co-pX are closely related conjugated polymer materials. This PFO easily produces excimers and dimers as evidenced by the steady state and ASE spectra [35]. However, PFO-co-pX does not exhibit such aggregation in a ground state or excited state as shown by the absorption, fluorescence, LIF, and ASE spectra (see Figure 2, Figure 3, Figure 4, Figure 5 and Figure 6). The only possible reason is that the co-polymer segment pX (2,5-p-xylene) has a strong tendency to go out of plane of PFO and sterically hinder association and aggregation in the ground or even in the excited state. Our hypothesis gains additional support from the spectral characterization of MEH-PPV and MEH-co-BEPH-PPV reported in our earlier papers [37].

**Figure 10:**
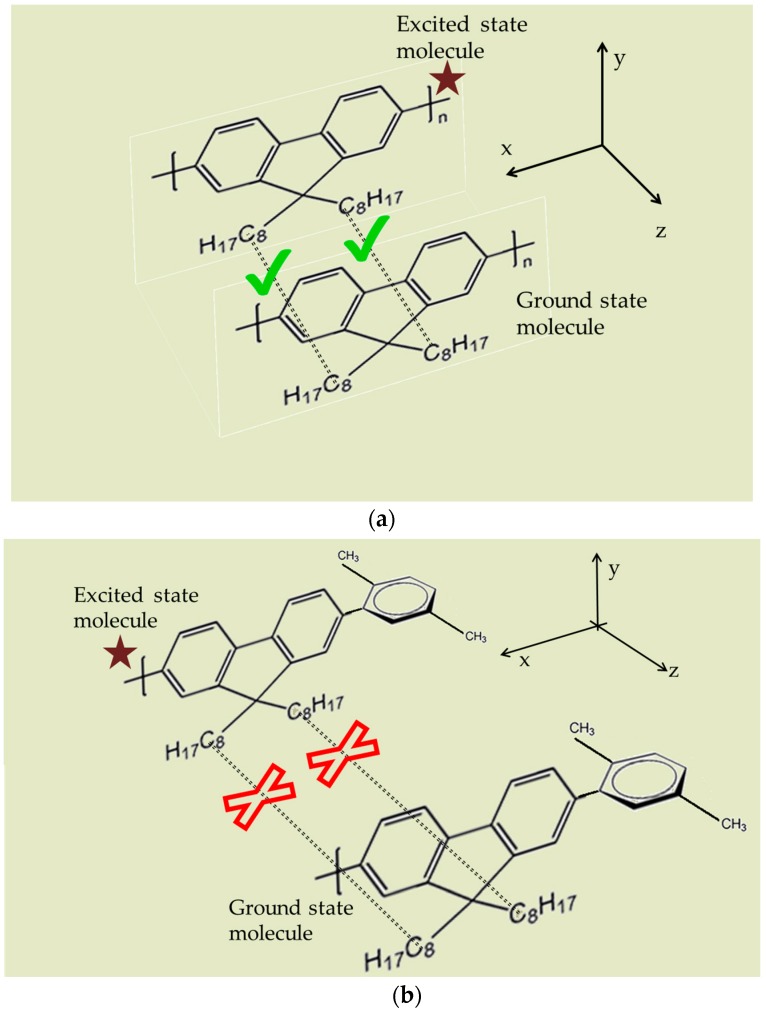
The plausible mechanism of formation (**a**) excimer in case of PFO alone; (**b**) the absence of any aggregation such as excimer in the case of PFO-co-pX—the pX segment forbids excimers by orienting in the XZ plane and sterically hindering the excimer formations.

Figure 10 shows the plausible mechanism of PFO and PFO-co-pX in the excited state. Figure 10a indicates that at least some of the ground-state and excited-state PFO still remain in the XY plane and manage to come together to form aggregation, with a sandwich configuration, either in a ground state, in an excited state, or both [35,39]. In contrast, such a close encounter is forbidden by the p-Xylene moiety which often orients itself in the XZ plane and sterically hinders the formation of any aggregation, as shown in Figure 10b.

MEH-PPV easily exhibits ASE from excimers and with some difficulty from double excimer. In fact, it was difficult to obtain ASE from the monomeric state of MEH-PPV. On the other hand, one can easily obtain ASE from the monomeric form of MEH only when tied up with the BEPH as a copolymer segment [37]. The copolymer segment usually orients out of plane with the MEH component and sterically hinders any aggregation. This is called intramolecular steric hindrance, a decisive factor in PFO-co-pX, too.

Even in the monomeric state, the present conjugated polymer material PFO-co-pX exhibits higher optical output, gain, etc. as the concentration is increased up to a certain critical level (from 60 to 90 μM). However, beyond a certain level (200–500 μM) excessive collision leads to quenching and rapid fall in intensity and optical gain as shown in Figure 8 and Figure 9.

## 5. Conclusions

In this preliminary report, study of the spectral and laser properties of a new conjugated polymer PFO-co-pX, absorption, and fluorescence and ASE spectra were taken. When this conjugated polymer was pumped by a high pump power laser at 355 nm, optical gain was so high to produce ASE as manifested by significant spectral, temporal, and spatial narrowing compared to LIF. The study has shown that this conjugated polymer has the best laser efficiency of 3% in toluene at a concentration of 90 μm and pump power of 10 mJ. Additionally, this is one of the very few conjugated polymer materials that does not exhibit any aggregation in a ground or excited state. This may be attributed to the steric hindrance of co-polymer segment p-Xylene.

## Figures and Tables

**Table 1:** Quantum yield of conjugated PFO-co-pX polymer in different solvents.

Solvent	Quantum Yield
Toluene	0.3
THF	0.29
Benzene	0.24

**Table 2:** Maximum ASE intensity values of conjugated (ADS145UV) polymer in different solvents at 200 μM, 12 mJ.

Solvent	ASE Intensity (Arbitrary Unit)
THF	1899
Benzene	3274
Toluene	9521
